# The PopGrouper as a tool for morbidity adjustment in regional comparisons of health care: an analytical framework

**DOI:** 10.1007/s43999-025-00068-y

**Published:** 2025-08-06

**Authors:** Anika Kreutzberg, Chrissa Tsatsaronis, Thomas G. Grobe, Wilm Quentin, Reinhard Busse, On behalf of the PopGroup consortium

**Affiliations:** 1 Department of Health Care Management, Technische Universität Berlin, Berlin, Germany; 2 Abteilung Gesundheitsberichterstattung und Biometrie, Institut für angewandte Qualitätsförderung und Forschung im Gesundheitswesen GmbH, Göttingen, Germany; 3 Department for Planetary and Public Health, Universität Bayreuth, Bayreuth, Germany

**Keywords:** Population classification, Regional analysis, Risk adjustment, Claims data, Germany

## Abstract

**Background:**

Analyzing regional variations can help improve equity, efficiency, and quality in health care provision. The PopGrouper is a population-based classification system which classifies persons with similar health care needs into distinct groups. It exhibits a high degree of morbidity differentiation. We present an analytical framework to use the PopGrouper in examining regional variations across different outcomes and populations using routine patient-level data.

**Methods:**

We develop a two-step empirical strategy to examine the relative regional performance on a set of efficiency and quality outcomes (e.g., hospital bed days, cost of care, mortality). First, we propose PopGroup-standardized observed-to-expected ratios to compare regional performance. Second, we develop a multilevel regression model to separately estimate regional variation related to patient need measured by the PopGroup and variation related to regional characteristics.

**Results:**

We provide an analytical framework that demonstrates the PopGrouper’s application as a tool for morbidity adjustment in the assessment of relative regional performance in efficiency and quality outcomes and the regional characteristics that explain this performance. We provide suggestions for empirical notation, interpretation of results, and graphical analyses of findings. The developed framework will be applied in subsequent empirical papers.

**Conclusion:**

This paper sets the analytical foundations to be applied in regional comparative analyses using the PopGrouper allowing for conclusions about unexplained variations in quality and efficiency of health care.

**Supplementary Information:**

The online version contains supplementary material available at 10.1007/s43999-025-00068-y.

## Introduction

Regional variations in health care utilization are undesirable differences in healthcare provision if they cannot be attributed to patient needs or preferences [1]. Reducing unwarranted variations in healthcare provision is an important health policy goal in order to improve equity, health system efficiency, patient-centeredness, and quality of health care provision [2].

A large body of literature aims to explore, quantify, and explain regional variations in various areas of health care [3]. A major challenge when studying regional variations is to distinguish unexplained variations from those related to specific characteristics of the resident population in the regions being compared. Factors that may influence these variations include differences in population structure (e.g., composition by age and gender) [4, 5], disparities in disease prevalence [6–8], and characteristics arising from socioeconomic status (e.g., income, employment status) and lifestyle [9, 10].

There are several ways to account for morbidity related regional differences. First, patient-level information (demographic and medical) can be used as predictors in regression analysis [11–14]. However, a meaningful quantification of multimorbidity is complex and studies use varying definitions of chronic conditions and differing numbers of condition categories [15]. Further, results depend on model choices and regression models are challenged when interactions between the relevant adjustment variables become more complex [16]. Second, generic morbidity measures using administrative data such as the Charlson Comorbidity Index [17] or the Elixhauser Comorbidity Measure [18] have been developed to measure morbidity in specific subpopulations such as hospitalized patients. These measures rely on weighted indices, organ- or system-based approaches, or clinical judgment with considerable variation in number of included conditions. The selection of conditions is most often based on prevalence rather than any impact on health care resource use [19]. Another option is to use population classification or segmentation systems based on claims data. These systems aim to categorize individuals of an entire population into mutually exclusive groups with each group representing a specific combination of demographic and diagnostic or medical attributes, which offers potential prognostic value for clinically relevant outcomes [20]. They can be either completely data-driven using statistical clustering methods [21] or they combine data-driven methods with clinical expertise to build clinically meaningful categories [20].

Population classification systems can serve in various areas to improve healthcare efficiency. For example, they can inform decision-making processes and support resource management and health care planning. In some health systems, they have already been proposed for capitation-based financing models [22]. They can also facilitate public health research by identifying trends and patterns in specific populations. Pioch et al. [23], for example, used a data-driven segmentation approach to derive cost profiles as well as morbidity and care patterns for the population in a German district. Existing classification systems are also used for morbidity adjustment in health services research, such as evaluating new care models [24], analyzing the impact of payment models on the risk of ambulatory care-sensitive hospital cases [25], or predicting healthcare expenditure [26, 27].

The PopGrouper is a population classification system developed for the German context which uses demographic and medical information from all sectors of health care within a given year from claims data [28–30]. It combines formal grouping rules which are based on medical expertise with data-driven classification and regression tree (CART) methods to build mutually exclusive groups which are both medically meaningful and homogeneous in terms of their health care resource utilization. As such, the PopGrouper exhibits a high degree of morbidity differentiation and considers multimorbidity. The basic ideas of the grouping algorithm are described in Supplementary Material 1.

In this paper, we demonstrate how the PopGrouper can contribute to improve the analysis of regional variation in health care. We present an analytical framework to use the PopGrouper as a tool to adjust for morbidity in examining regional variations in efficiency and quality outcomes. We describe in general terms the empirical approaches and provide guidance on the interpretation and graphical demonstration of findings. The empirical articles in this collection of Research in Health Services & Regions build on this common analytical strategy to examine regional variation using various study populations as examples.

## Methods

### Data and study populations

All analyses use claims data from a large German sickness fund (BARMER) for the years 2022/2023^1^. We consider patients being diagnosed or treated in 2022 in inpatient or outpatient settings with one of five disease conditions which are listed in Table 1. The detailed selection criteria for each study population can be found in Supplementary Material 2. Except for acute stroke, we deliberately defined broad and heterogeneous study populations to demonstrate the PopGrouper’s ability to adjust for this heterogeneity.

**Table 1:**
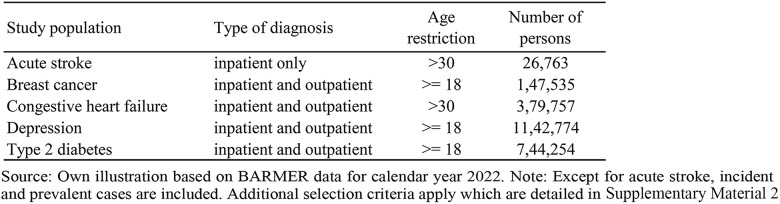
Size of study populations and number of PopGroups

We chose these conditions as examples to demonstrate the application of the PopGrouper in regional analyses as they are characterized by high prevalence and impact on the population, significant health-economic importance, and the risk of being affected by unwarranted regional variations [31–35]. The study population size varies between 26,763 persons with acute stroke and 1,142,774 with depression. Out of 776 possible PopGroups (version 1.0), the number of PopGroups in each study population ranges between 240 (acute stroke) and 677 (depression) (Table 1).

For each study population, we consider a set of outcome variables presented in Table 2 which reflect efficiency and quality aspects of health care. All outcomes are measured for the year 2023.^2^ Both, cost and the number of hospital bed days can be considered as measures of resource use in health care [36, 37]. Hospitalization particularly due to type 2 diabetes or congestive heart failure as ambulatory care sensitive conditions are considered potentially avoidable given sufficient primary health care provision. Regional variation in avoidable hospitalizations may therefore be an indicator of poor ambulatory treatment quality [12, 38]. Mortality is a generic patient-relevant measure of treatment quality and also often used in regional comparisons of performance [13, 14]. Further, we investigate a range of disease-specific outcomes which we consider quality indicators in the treatment of the specific disease and have been analyzed in the context of regional comparisons elsewhere such as amputations with type 2 diabetes [39], treatment of breast cancer and stroke in certified hospital units [40, 41], or participation in disease management programs which have been developed to reinforce guideline adherence and improved quality of care for patients with chronic conditions [42].

**Table 2:**
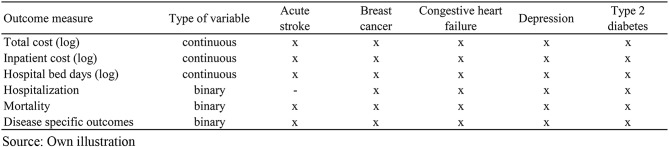
Outcome measures by study population

As regional unit of analysis we use the 96 spatial planning regions in Germany (referred to as regions in the following) defined by the German Federal Institute for Research on Building, Urban Affairs and Spatial Development. They are almost identical to the planning regions of the federal states. The region is defined by the patient’s place of residence which may not be the region the patient was treated in. Table 3 displays the range across the 96 regions regarding the number of persons, the number of persons per 1,000 insured, and the average PopGroup severity score in each study population. The PopGroup severity score reflects the relative complexity and severity based on the standardized mortality ratio, average healthcare utilization (hospital days and outpatient cases), and average costs associated with that group (see also Supplementary Material 1). In the PopGrouper version 1.0, the score ranges from − 0.4 to 13.1.

**Table 3:**
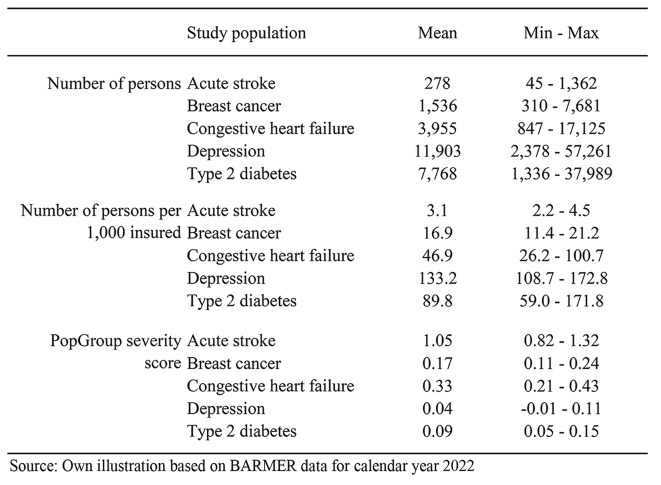
Patient characteristics (mean; min/max) across 96 regions

**Fig. 1:**
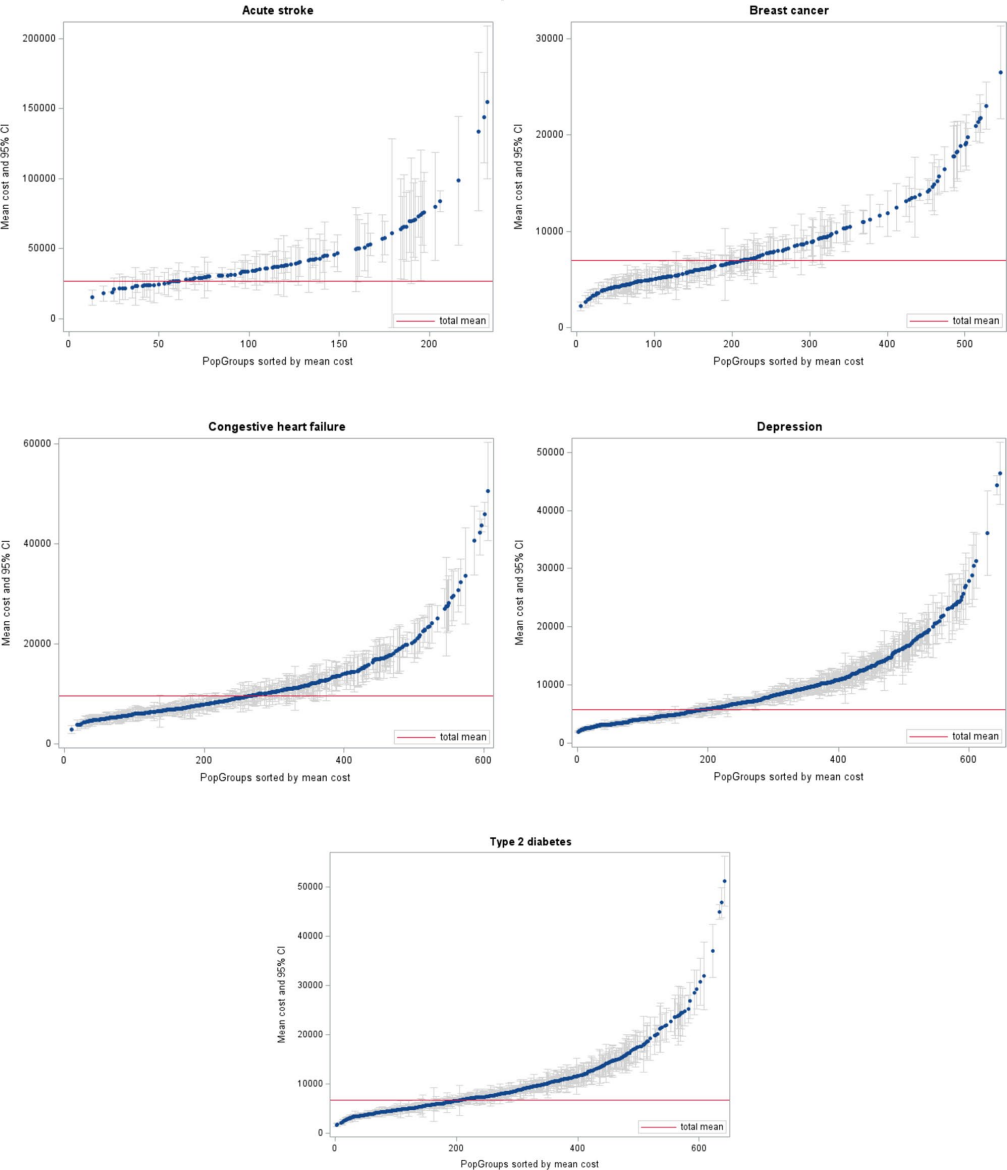
Distribution of total cost across PopGroups in the five study populations. Source: Own illustration based on BARMER data with PopGroup classification in calendar year 2022 and total costs in Euros per person measured in calendar year 2023. Note: For better graphical representation, only PopGroups with at least 100 persons are displayed (at least 10 persons for the acute stroke population)

As can be seen in Table 3, the average severity score varies between regions in each study population which indicates that the PopGroup distribution in each region varies as well. At the same time, we observe large variations in the selected outcomes by PopGroup in each study population. For example, Fig. 1 illustrates the wide variation of total costs across PopGroups in each of the five study populations.

From this, we assume that the region-specific PopGroup distribution impacts the region’s outcome performance and hence adjusting for this PopGroup distribution is important to draw conclusions about relative regional performance. In the following, we propose an analytical framework to conduct these adjustments using the PopGrouper.

### Analytical framework

The proposed empirical strategy builds on two main components: (i) observed-to-expected ratios standardized by the PopGroup distribution in each region, and (ii) a multilevel regression model to estimate the level of variation in outcomes attributable to the patient’s PopGroup and the level of variation attributable to the region (as well as to observed regional characteristics).

#### Observed-to-expected ratios

One of the strengths of the PopGrouper is that every patient is assigned to exactly one PopGroup (categorical model [16]) and each PopGroup combines several patient-level characteristics (such as morbidity, age, level of care dependency). This allows us to use the PopGrouper as a tool to indirectly standardize regional performance by those patient characteristics and calculate observed-to-expected ratios (O/E ratios). The O/E ratio is a widely used and easy to interpret concept to compare performance between regions or providers [43]. It allows adjusting by building the ratio of the observed number of events for a given outcome variable in a region and the expected number of events according to a reference population. The reference population is often the total population including all regions [44].

Similarly to the commonly used age and sex standardized mortality ratio (SMR), we first define the age-sex-standardized outcome ratio (ASOR_j_) for each region as follows:

1 $$ASO{R_j} = {Y_{j;O}}/{Y_{j;E}}$$

Where $\:{Y}_{j;O}$ is the observed outcome in region $\:j=1\dots\:.J$ and $\:{Y}_{j;E}$ as the expected outcome is defined as:

2 $${Y_{j;E}} = \Sigma _{ASG = 1}^n\{ {N_{j;ASG}}*{Y_{r;ASG}}\}$$

with $\:{N}_{j;ASG}$ representing the number of patients in region j in a specific age and sex group (ASG), and $\:{Y}_{r;ASG}$ being the age-sex-specific outcome in the reference population. The reference population is the national study population.

We translate this approach into a PopGroup standardized outcome ratio

3 $$PGSO{R_j} = {Y_{j;O}}/{Y_{j;E}}$$

where $\:{Y}_{j;E}=\sum\:_{PG=1}^{n}\left\{{{N}_{j;PG}*Y}_{r;PG}\right\}$ with $\:{N}_{j;PG}$ reflecting the number of patients in region j in a specific PopGroup (PG), and $\:{Y}_{r;PG}$ being the PopGroup-specific national mean of the outcome. That means, an expected value for each region based on its specific PopGroup distribution is calculated. We calculate $\:{ASOR}_{j}$ and $\:{PGSOR}_{j}$ for each outcome in each study population and compare to which extent regional performance varies between the two adjustment approaches and describe best and worst performing regions by observed regional characteristics and utilization patterns.

#### Multilevel regression analysis

Another approach to explore PopGroup-adjusted regional variation in quality and efficiency outcomes is to specify a multilevel regression model (MLM) which recognizes that patients (level 1) are clustered within regions (level 2). The multilevel structure allows estimating variations at each level of the hierarchy and thus analyzing regional effects within the models as a two-stage process [45, 46].

The regression model takes the following general form:

4 $${y_{ij}} = {\beta _{0j}} + {\gamma _{01}}P{G_{ij}} + {\varepsilon _{ij}}$$

where $\:{y}_{ij}$ is the outcome and $\:{PG}_{ij}$ is the PopGroup of patient i $\:(i=1,\:\dots\:,\:I)$ in region j $\:(j=1,\:\dots\:,\:J)$ which is entered as a set of dummy variables with the reference group being the most populated PopGroup in each study population. The regional-level intercepts $\:{\beta}_{0j}$ are treated as random intercepts defined as a combination of a fixed grand mean $\:{\gamma}_{00}$ and a deviation from that mean $\:{u}_{0j}$:

5 $${\beta _{0i}} = {\gamma _{00}} + {u_{0j}}$$

Equations (4) and (5) can be combined into a random intercept model [47]:

6 $${y_{ij}} = {\gamma _{00}} + {u_{0j}} + {\gamma _{01}}P{G_{ij}} + {\varepsilon _{ij}}$$

The term $\:{\gamma}_{01}{PG}_{ij}$ is the fixed effect of the PopGroup at the patient level. The random variable ${u}_{0j}\sim{N}(0, {\tau}^{2})$ represents the random intercept variance at the regional level (level 2), and the error term ${\varepsilon}_{ij}\sim{N}(0, {\sigma}^{2})$ is the level 1 variance component. The regional-level intercepts $\:{\beta}_{0j}$ measure the differences in outcomes between regions, controlling for patient case-mix expressed by the PopGroup.

As we find the distribution of costs and number of hospital bed days to be positively skewed, we convert both outcome measures into logarithmic form to normalize the overall distribution of outcomes. We then estimate Eq. (6) using log-linear random intercept models.

For our binary outcomes, we use hierarchical generalized linear models (HGLM) to estimate Eq. (6) for the different study populations. HGLMs allow for the transformation of the outcome using a nonlinear link function and the choice of the appropriate non-normal error distribution so the model building strategies and the interpretations used for hierarchical linear models are still applicable [48, 49]. We use the binomial distribution and the logit link to estimate Eq. (6). In this case, the estimated $\:{\widehat{\gamma\:}}_{01}$ represents the log odds of the outcome being 1 (e.g. being hospitalized) for a given PopGroup. For a more intuitive interpretation, we report predicted probabilities ($\:{\theta}_{ij}$)

of the outcome being 1 when describing the relationship between PopGroup and binary outcomes as:

7 $${\theta _{ij}} = \frac{{{e^{yij}}}}{{1 + {e^{yij}}}}$$

In Eq. (6), regional effects are treated as random intercepts only. In a first step, we use these estimation results to compare the relative performance in outcomes between regions. In a second step, we add random effects (intercepts and slopes) for a series of observed regional variables $\:{Z}_{j}$. For this purpose, we modify our regression equation as follows:

8 $${y_{ij}} = {\gamma _{00}} + {u_{0j}} + {\gamma _{01}}P{G_{ij}} + \left( {{\gamma _{10}} + {u_{1j}}} \right){Z_j} + {\varepsilon _{ij}}$$

or

9 $${y_{ij}} = {\gamma _{00}} + {u_{0j}} + {\gamma _{01}}P{G_{ij}} + {\gamma _{10}}{Z_j} + {\varepsilon _{ij}}$$

The vector $\:{Z}_{j}$ represents a set of regional characteristics including demographic factors (average population age, population density), supply-side factors (general practitioners per 10,000, inpatient beds per 1,000), level of urbanization, and level of socioeconomic deprivation measured by the German Index of Socioeconomic Deprivation [50, 51]. The term $\:{\gamma}_{10}{Z}_{j}$ represents the fixed effect (slope) of $\:{Z}_{j}$ at the regional level which is allowed to vary with the random slope component $\:{u}_{1j}{Z}_{j}$. The random intercept and slope model defined in equations (8) and (9) assumes variability in the baseline of the outcome of different regions (random intercept) and allows for the incremental effects of $\:{Z}_{j}$ to be different for each region (random slope).

A challenge of using a highly differentiated classification system such as the PopGrouper in regression analysis for regional comparisons is the risk of small sample size per PopGroup and region. In multilevel modelling, the number of clusters matters more than the number of observations per cluster [52]. Simulation studies showed that a minimum of 50 to 100 level 1 units and 40 to 80 level 2 units are needed to accurately estimate fixed effects depending on the size of the intercept variance [53, 54]. For our analysis, we chose to include PopGroups with a population size of at least 1% of the total study population. For persons in PopGroups with less than 1% of the study population, we use the higher aggregated 10 Macro PopGroups each person is allocated to instead of the PopGroup.

### Graphical analysis

Regional variation based on the PopGroup-standardized outcome ratios can be graphically visualized to detect patterns and support the comparison of relative regional performance between outcomes and study populations. We present distribution diagrams in which the 96 regions are sorted by the O/E ratio. The reference line at 1 represents the national average. Regions plotted below the reference line represent regions with better outcome performance than the national average and vice versa.

The distribution of regions by their relative performance can then be compared visually among the different O/E ratio measures (crude rate, age-sex-standardized, PopGroup standardized and PopGroup adjusted based on regression results) to elaborate how the relative regional performance changes depending on the type of standardization applied.

## Expected results

### Interpreting PopGroup standardized outcome ratios

The PGSOR represents a measure of relative regional performance as it indicates the relative position of the region compared to the national average after considering the distribution of PopGroups in the region-specific population. If the PGSOR is greater than one, the region exhibits an outcome value above the national average and vice versa. Table 4 presents exemplary PGSOR for the outcome hospitalization in the type 2 diabetes population for the 10 regions with the smallest (Top10) and the largest (Bottom10) PGSOR each. Region 769 has the smallest PGSOR of 0.43 resulting from 9 observed persons with a hospitalization in 2023 and 21 persons which would have been expected to be hospitalized according to the region’s PopGroup distribution. In contrast, region 803 has the largest PGSOR of 1.47 (74 observed hospitalized persons compared to 50 expected persons) indicating a 47% higher hospitalization compared to the national average. This form of standardization allows for a uniform presentation of results across different outcomes and study populations. Assuming that a patient’s treatment outcomes are associated with the patient’s level of morbidity and health care utilization, which is reflected by the PopGroup, the $\:PGSOR$ can be interpreted as a morbidity-adjusted comparison of regional performance.

**Table 4:**
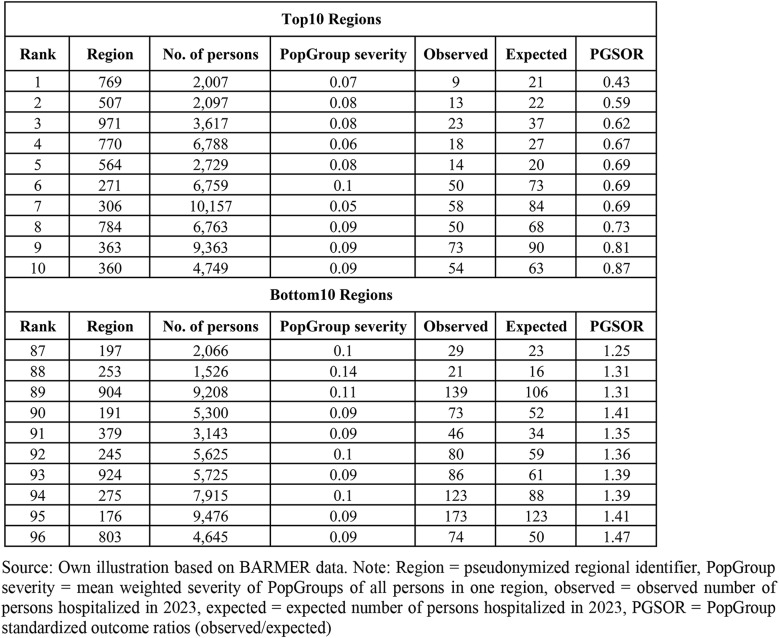
Exemplary results of PopGroup standardized outcome ratios (PGSOR) for hospitalization with type 2 diabetes

**Fig. 2:**
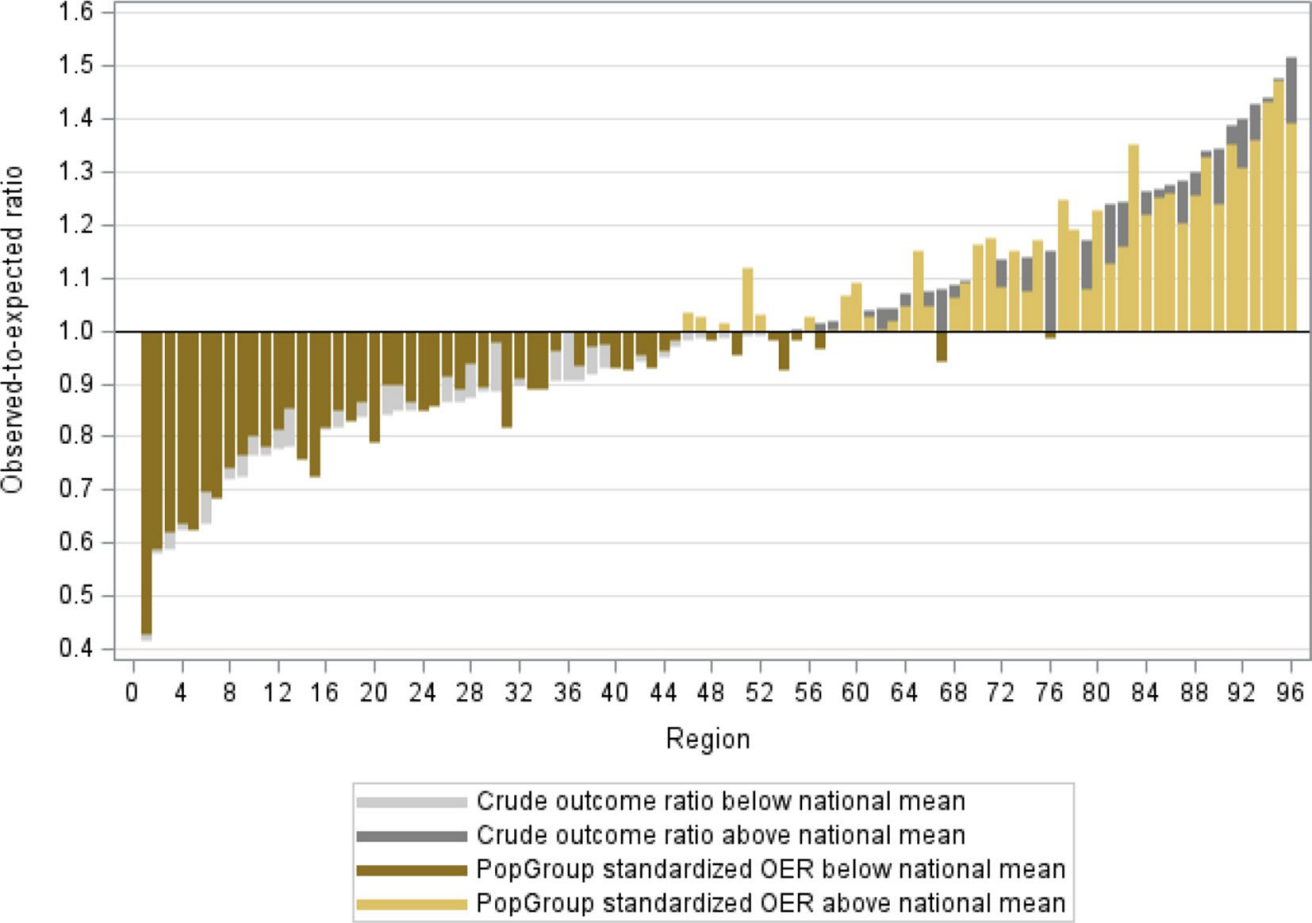
Exemplary distribution diagram of PopGroup standardized outcome ratios (PGSOR) for hospitalization with type 2 diabetes. Source: Own illustration based on BARMER data

Figure 2 displays the distribution diagram for the PGSOR of the outcome hospitalization in the type 2 diabetes population. The reference line at 1 represents the national average. Regions are ranked by the crude outcome ratio without standardization which is presented by the grey bars. The yellow bars plot the PGSOR in comparison. Larger yellow bars compared to the grey bar in the background indicate regions with a larger difference in outcome performance after PopGroup standardization and vice versa. Some regions switched from below average to above average outcome performance after PopGroup standardization (darker yellow bars in the lighter yellow group).

### Interpreting the multilevel modeling results

The multilevel modeling approach allows us to disentangle the within-cluster effects (the extent to which the PopGroup is associated with the outcome) from the between-cluster effect (the extent to which regional characteristics are associated with the outcomes). The regression analyses for each study population and outcome provides insights into three broad sets of issues.

First, we are able to explore the relationship between PopGroups and outcomes at the patient-level. Estimating $\:\widehat{{\gamma\:}_{01}}$ from Eq. (6) reveals insights into the relative impact of the PopGroup on the outcome. The predicted outcomes can be interpreted as the average outcome in one PopGroup compared to the reference PopGroup (the most populated PopGroup in each study population). We also quantify the explanatory power the PopGroup has for each of the outcomes or, in other words, the proportion of total variation that is explained by patient-level characteristics represented by the PopGroup. We assess whether this explanatory power remains generally consistent across outcomes and study populations. For this, we analyze the adjusted R2 statistics as the most common performance measure for continuous outcomes [55]. For the binary outcomes, we use Nagelkerke’s R2 which is often used for generalized linear models [55, 56].

Second, we explore the level of regional variation based on the regression results and compare between outcomes and study populations. We use intraclass correlation coefficients (ICC) to show the between-cluster variation by outcome and study population. The ICC is conceptually comparable to the R2 effect size from regression analysis [46]. The ICC is calculated using the estimated random intercept variance $\:{\tau\:}^{2}=var\left({u}_{j}\right)$ and the level 1 variance $\:{\sigma\:}^{2}=var\left({\epsilon}_{ij}\right)$ from the unconditional means model (i.e. containing no predictors):

10 $$\:ICC=\:\:\tau\:^2/(\tau\:^2+\:\sigma\:^2\:)$$

**Fig. 3:**
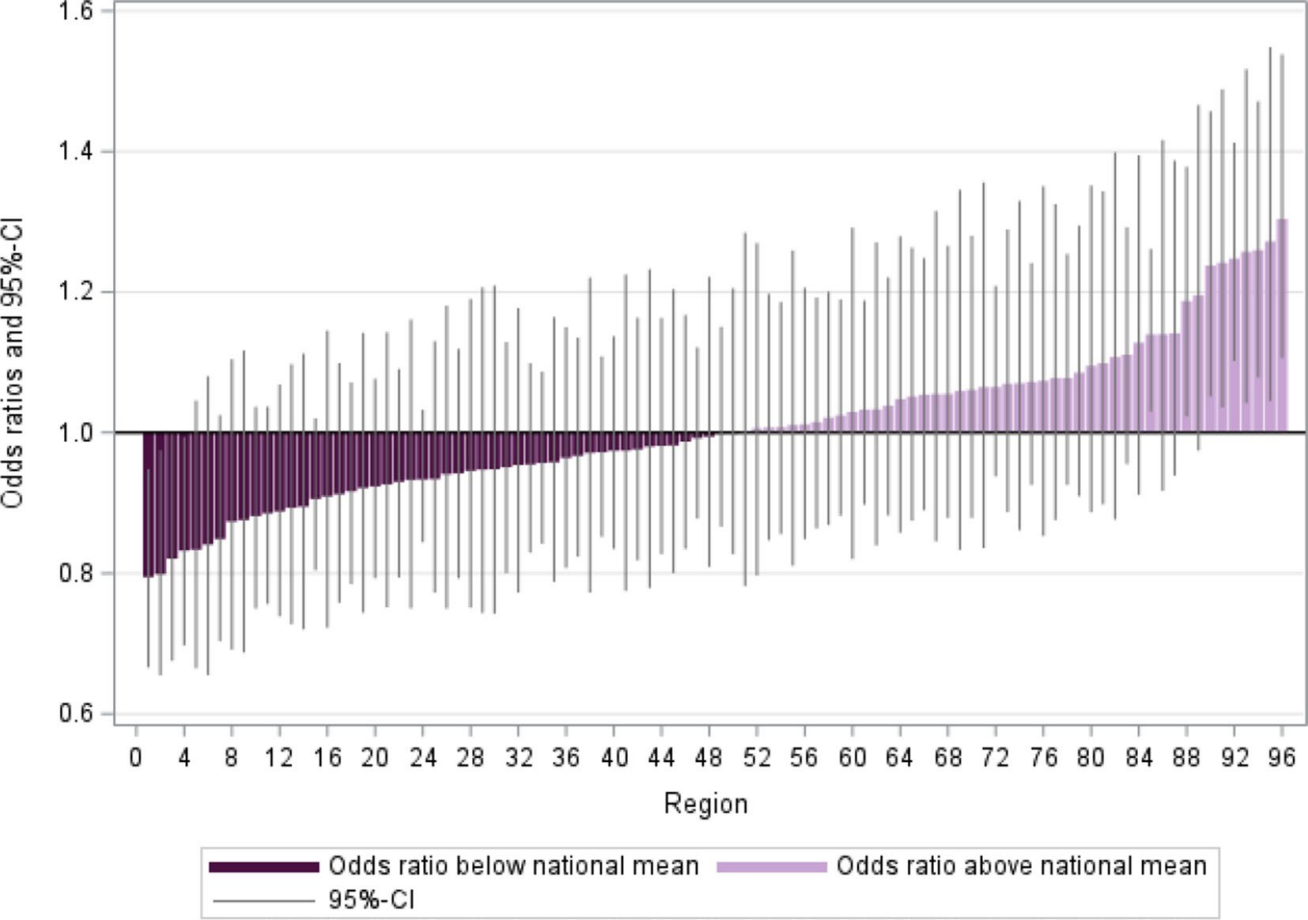
Exemplary distribution diagram of odds ratios for hospitalization with type 2 diabetes odds ratios. Source: Own illustration based on BARMER data

For binary outcomes, there is assumed to be no error at level 1 in hierarchical models. Therefore, we assume the level 1 variance component to be $\:{\sigma\:}^{2}={(\pi\:}^{2}/3)\approx\:3.29$ which refers to the standard logistic distribution [48, 52]. We interpret the ICC as the proportion of total variation in the outcome that can be attributed to the regional level independent from the PopGroup distribution in the region [11]. An ICC of zero indicates perfect independence between regions, whereas an ICC of one indicates perfect interdependence between regions [52]. In social research studies, ICC values between 0.05 and 0.20 are common in cross-sectional MLM applications [46]. Further, we can use the regression results from Eq. (6) to estimate PopGroup adjusted regional effects on the outcomes and compare the performance between regions using the region-specific intercepts. For the binary outcomes, we obtain the odds ratio for each region by exponentiating the estimated value of the regional effect and interpret this as measure of regional performance relative to the average of all [47]. As an example, Fig. 3 displays the estimated odds ratios (and confidence limits) of the outcome hospitalization in the type 2 diabetes population in a distribution diagram. Again, the reference line at 1 represents the national average. The odds ratio can be interpreted similarly to the O/E ratio with values greater than one (lighter purple) indicating a regional performance higher than the average and vice versa (subject to sampling errors).

For the continuous outcome variables, we calculate PopGroup-adjusted O/E ratio following Eq. (3) but using the estimated region-specific outcomes based on the regression analysis. Third, we explore the influence of specific regional-level characteristics on outcomes by interpreting the fixed effects coefficients of the level 2 variables included in vector $\:{z}_{j}$ in Eq. (8). From the regression results we can conclude which observed regional factors (e.g. hospital bed supply, number of physicians, level of urbanization or socio-economic deprivation) have a significant impact on regional performance in the outcomes after accounting for the PopGroup distribution in the regions.

## Discussion

This article provides a conceptual and empirical framework to use population classification systems such as the PopGrouper as a tool to adjust for patient-level characteristics in regional analyses. It bridges the gap between literature that uses different methods of adjusting for patient-related factors to explain regional variations [11–14] and literature that uses population classification systems for morbidity adjustment in health services research [24–27]. Our suggested approach contributes to this body of literature in several aspects:

To start with, the PopGrouper provides some important advantages as a tool to adjust for morbidity related patient needs in regional comparisons, compared to other traditionally used measures for morbidity adjustment. Morbidity measures should have clear criteria for selecting conditions that impact patient outcomes or health care resource utilization, account for condition severity, and use accessible routinely collected data [15]. The PopGrouper provides a sophisticated algorithm based on both clinical expertise and empirical algorithms to deal with multimorbidity that goes beyond simply counting and adding diagnoses. It combines patient-level diagnostic or medical information into groups which offer prognostic value for clinically relevant outcomes and health care utilization. It is designed specifically for comparing health outcomes at a population level rather than among specific clinical patient cohorts and considers the very ill but also those with little or no health care utilization. The PopGrouper builds on routinely collected claims data from all settings of health care (e.g., inpatient, outpatient, ambulatory, rehabilitation, long-term care, medication, remedies and aids) including all providers, diagnoses, and other available health information of a whole calendar year. With the transparent grouping algorithm, the PopGrouper presents a standardized instrument which allows to describe health care needs of various populations across conditions, regions, and potentially, countries.

Next to this, our proposed analytical framework adds a number of contributions. First, we present a simple way of indirectly standardizing observed-to-expected ratios by morbidity using the PopGrouper. The PopGroup standardization allows for a uniform presentation of results across different outcomes and study populations (and potentially across countries). It further provides an intuitive interpretation of results which can be supported by the suggested graphical visualizations. This simple but effective approach can help make research findings on regional comparisons more accessible for health policy makers and non-academic audiences (e.g., informing the general population).

Second, we add a more sophisticated approach of using the PopGrouper for regional analyses using multilevel regression analysis. This approach allows us to separately explore (i) the PopGroup effect on outcomes and the PopGroups’ explanatory power, (ii) the relative regional performance on outcomes independent from the region’s PopGroup distribution, and (iii) the impact of selected regional characteristics on the estimated regional differences. We transform the regression results into observed-to-expected ratios using the same simple interpretation and graphical visualization as in the first approach to make the regression results easily accessible as well. The multilevel modelling further helps us to account for the fact that patients are clustered within regions which violates the assumption of independent observations in linear regression.

Third, the empirical approach presented makes use of routine patient-level data in comparison to other regional analyses which often rely on aggregate or average regional data which are limited to a particular setting of care [12, 14]. The analysis of patient-level data is both more robust and insightful in the analysis of variations in health care utilization [36, 37]. It allows for large sample sizes and uses information on a patient’s health care utilization over time going beyond a single inpatient stay or ambulatory treatment. As the shift from inpatient to ambulatory health care delivery becomes more important, this is an increasingly important issue to address in regional comparisons [57].

Besides the advantages outlined above, some aspects need to be considered when interpreting results based on the suggested empirical approach. First, the analysis builds on the PopGrouper as an instrument to account for morbidity related healthcare resource use. A number of grouping and segmentation approaches exist internationally, both completely data-driven or combined with medical expertise, and all of them come with their strengths but also limitations [20, 21]. Most of these tools perform reasonably well in predicting healthcare expenditure and identifying high-cost patients [58]. However, the ideal segmentation approach has yet to be identified. The PopGrouper is a new tool that still has to prove its specific advantages over other tools, which goes beyond the scope of this paper. Some work is already in progress to be published elsewhere but future research is needed to extend the knowledge about the PopGrouper’s applicability in various applications. For example, the temporal stability of the PopGrouper still has to be tested in longitudinal studies to demonstrate its potential for monitoring regional variation over time in a standardized and comparable manner. Also, the highly differentiated grouping system challenges regression analysis if case numbers per PopGroup and region are small. We propose to aggregate PopGroups to larger Macro PopGroups as a solution, risking loss of information. Future research should elaborate the ideal balance between unit size and level of information utilized.

Second, related to the previous aspect, the database used for both the development of the grouping algorithm and its application in regional analyses must be critically assessed. Analyses are based on one single health insurance fund which may limit generalizability of results. However, the BARMER health insurance fund covers about 10% of the German population (over 8 million persons in 2022) [59]. As one of the largest statutory health insurance funds in Germany it has shown to be sufficiently large to reflect the structure of the total statutory health insurance population [60]. Nevertheless, differences, particularly in the regional distribution of insured persons and morbidity burden, may exist, as can be assessed using the interactive morbidity and social atlas published by the research institute of BARMER [61]. The PopGrouper will be released as open-source algorithm and made freely available to the public. So going forward, the grouping algorithm can be applied to other datasets and populations to test representativity. Next to considerations regarding generalizability of results, general limitations when using administrative data apply. Particularly, in this context the lack of detailed clinical information such as disease severity or functional status compromises the accuracy of morbidity adjustment. Potential biases may also arise due to the primary purpose of data collection being billing rather than research [62]. For example, variability in coding practices and financial incentives may have affected diagnosis reporting [63].

Third, it needs to be noted that we define “region” as the region the patient is living in. This may be different from the region the treatment has taken place in [64–66]. Therefore, the regional performance measured needs to be interpreted as the level of efficiency or quality of care received by the population living in this region rather than the efficiency or quality of health care providers in this region. However, this perspective is relevant for policy makers or planners concerned with assuring health care provision for a particular population. Second, in our approach regional performance is defined as a relative measure compared to the national mean. That means the national average is used as the benchmark to compare regions against. There are two main reasons behind this conceptual choice. On the one hand, normative benchmarks would need information on the correct or desired outcome level (e.g. the correct number of hospital bed days for a stroke patient). In most cases, those normative values are unavailable and would require complex procedures to be established. This is particularly true when analyzing more generic outcomes (such as costs, hospital bed days, mortality) where normative values – if existing – are limited to specific subpopulations. On the other hand, using the national average as comparator brings the advantage of focusing the interpretation of the results primarily on the extent of regional deviations, rather than on the discussion of any chosen normative value. However, this choice limits the interpretation of results regarding potential over- or under-provision since below-average regional performance can mean either more efficient provision or under-provision, and vice versa. We try to address this by looking at both efficiency and quality outcomes and comparing regional performance across these outcomes to explore whether regions with below-average costs have better or worse quality outcomes (e.g., mortality). At the same time, the overall level of performance in Germany may be relatively high or low when compared internationally. In such cases, even significant deviations from the average would need to be interpreted against this benchmark. When comparing costs at regional level it needs to be noted that these may be influenced by varying prices negotiated at the federal level. However, these differences are expected to be small and negligible in this context.

Finally, the approach builds on the assumption that the PopGrouper captures patient health care needs. As outlined above, the PopGrouper utilizes a large amount of medical information over time to approximate patient needs. Nevertheless, patient needs are a complex concept and their measurement using the PopGrouper will be imperfect but potentially closer than other measures available [67]. At the same time, the PopGrouper was not primarily designed to predict and extrapolate future health care outcomes.

To conclude, we developed a generic analytical strategy to examine regional variations in health care that can be applied to a diverse set of efficiency and quality outcomes and different study populations and contexts. The proposed strategy allows (i) to quantify the explanatory power of PopGroups on treatment outcomes, (ii) to assess relative regional performance in health care efficiency and quality, and (iii) to explore regional characteristics not related to patient-mix that explain this performance. Our approach can serve as a step towards standardizing research in regional comparison and thus help foster comparability of research findings across studies. This may help to draw more reliable conclusions regarding the generalizability of research output which in turn may reduce uncertainty of health policy decisions relying on these research findings.

## Electronic supplementary material

Below is the link to the electronic supplementary material.

Supplementary Material 1

Supplementary Material 2

## Data Availability

The data utilized in this current study are health insurance claims data provided by the German statutory health insurance fund BARMER. Due to legal and privacy restrictions, these data are not publicly available. Access to the data is subject to approval by BARMER and may require additional agreements regarding data protection and confidentiality.

## Footnotes

1 BARMER covers approximately 10% of the German population.

2 For the acute stroke population outcomes are measured for 365 days from the initial stroke event.
