# Supplementary material for: The PopGrouper as a tool for morbidity adjustment in regional comparisons of health care: an analytical framework
Source: Res Health Serv Reg. 2025 Aug 6;4:10. doi: 10.1007/s43999-025-00068-y (PMC12325817; doi:10.1007/s43999-025-00068-y)
Supplement: Supplementary file 1 — Supplementary Material 1 [file 43999_2025_68_MOESM1_ESM.pdf]

The PopGrouper version 1.0

The PopGrouper is a population classification system developed for the German healthcare context. It classifies individuals into medically meaningful groups (PopGroups) with similar levels of healthcare resource utilization (medically and economically homogeneous) taking into account all coded diagnoses within one calendar year. The grouping algorithm for the PopGrouper 1.0 is based on medical expertise and empirical analyses of claims data from the German statutory health insurance fund BARMER with over 8 million insured persons in the year 2022. Detailed grouping rules are published by Braun et al. [1]. The PopGrouper represents a categorical model in which each individual is assigned to exactly one PopGroup.

The complex grouping algorithm can be broken down in two main parts: (1) reducing complexity by grouping diagnoses into morbidity groups, (2) building distinct groups that are medically and economically meaningful. Figure 1 provides a simplified overview of the grouping process.

1.1. Part I: Reducing complexity in diagnoses by morbidity groups

1.1.1. Building Macro Disease Groups (MDGs) and Consolidated Disease Groups (CDGs)

In a first step, the over 14,500 existing ICD-10 codes of all insured persons in 2022 were reduced into medically meaningful groups which summarize several diagnoses. As a starting point, diagnostic groups (DxGs) which were developed for the morbidity-related risk adjustment in allocating budgets to health insurance funds in Germany (Morbi-RSA) were built. The selection criteria for the diagnostic groups defined by the German Federal Office for Social Security are updated regularly and can be accessed freely [2]. Depending on the disease, persons require either an inpatient or outpatient diagnosis, and possibly other additional criteria such as medication, or minimum length of treatment to be assigned to a certain diagnostic group. For example, the DxG 838 “Recurrent major depression” requires an inpatient diagnosis or a confirmed outpatient diagnosis in at least two quarters of the year with ICD-10 codes F33.2-3, at least one simultaneous medication prescription and a minimum treatment length of 10 days. As comparison, the DxG 105 “Type 1 diabetes mellitus with diabetic ketoacidosis or coma” only requires an inpatient diagnosis with ICD-10 codes E10.0, E10.01, E10.1 or E10.11.

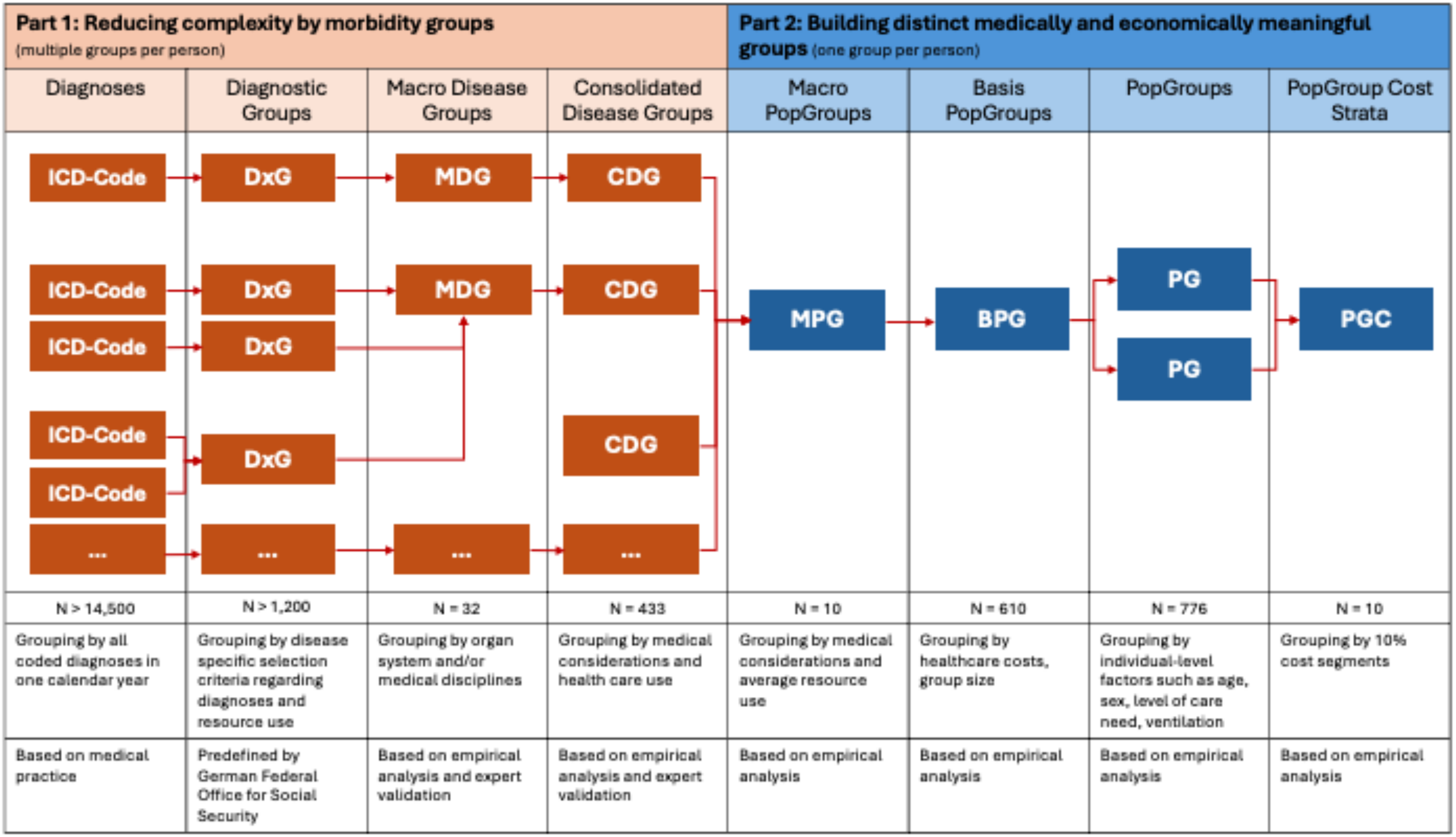

Figure 1: Simplified overview of the PopGrouper 1.0 grouping process

Source: Own illustration based on Tsatsaronis et al. [3].

The over 1,200 DxGs were grouped into 32 overarching Macro Disease Groups (MDGs) based on the affected organ systems and the medical disciplines involved in the treatment of the diseases (Table 1). Within each MDG, the DxGs were grouped into a total of 433 Consolidated Disease Groups (CDGs) based on predefined criteria. These CDGs are intended to group diagnoses into clinically meaningful categories, focusing on the nature of the disease and related care needs, regardless of medication, cause, or age. An individual may be assigned to several different CDGs. The resulting CDGs were validated by medical experts. A complete list of all 433 CDGs is published by Tsatsaronis et al. [3].

Table 1: List of Macro Disease Groups (MDG)

MDG	MDG name	MDG	MDG name
1	Infections	17	Vascular and circulatory system diseases
2	Neoplasms	18	Lung diseases
3	Diabetes mellitus	19	Urogenital system diseases
4	Metabolic disorders	20	Pregnancy, childbirth, and the puerperium
5	Liver diseases	21	Skin diseases
6	Gastrointestinal diseases	22	Burns
7	Musculoskeletal system disorders	23	Injuries
8	Blood disorders	24	Complications
9	Cognitive disorders	25	Transplants
10	Alcohol and drug abuse	26	Eye diseases
11	Mental disorders	27	Neonatal diseases
12	Developmental disorders	28	Chronic pain
13	Spinal diseases and injuries	29	Ear, nose, and throat diseases
14	Neurological diseases	30	Nutrition-related diseases and poisonings
15	Heart diseases	31	Gynecological and reproductive disorders
16	Cerebrovascular diseases	32	Andrological and reproductive disorders

Source: Translated from Tsatsaronis et al. [3].

1.1.2. Severity scoring of CDGs

To categorize or hierarchize different diseases across diagnoses in terms of their significance, a CDG severity score was developed based on three dimensions:

1. Medical severity, measured by standardized mortality ratios
2. Economic burden, measured by average total costs
3. Healthcare need, measured by average outpatient visits and inpatient days

A standardized composite severity score (z-score) was calculated from these components. Within the healthcare need dimension, inpatient service use was weighted twice in comparison to outpatient service use, assuming that hospitalizations reflect more severe conditions. All CDGs

were ranked by severity and assigned to one of five categories: very severe, severe, moderate, mild, or very mild.

1.2. Part II: Building distinct medically and economically meaningful groups

Based on the morbidity information from the MDGs, CDGs, severity levels, and other characteristics, distinct groups were built at different aggregation levels. Each insured person is assigned to exactly one group at each aggregation level.

1.2.1. Building Macro PopGroups (MPGs)

At the highest aggregation level, individuals were assigned to one of ten Macro PopGroups (MPGs) (Table 2). These are primarily based on medical considerations and average resource use. They represent either specific need groups (such as newborns or pregnancy, childbirth and postpartum period) or population segments with varying degrees of morbidity severity. For example, MPG 03 “Severe high-cost cases” includes individuals with at least one condition that is both very severe and among the top 20% of the costliest conditions. MPGs 05 to 08 are assigned based on a person’s highest CDG severity score.

Table 2: List of Macro PopGroups (MPG)

**MPG**	**MPG name**
01	Newborns
02	Pregnancy, childbirth and postpartum period
03	Severe high-cost cases
04	Acutely treated malignant neoplasms
05	At least one severe condition
06	At least one moderate condition
07	At least one mild condition
08	At least one very mild condition
09	Healthcare utilization without illness
10	No healthcare utilization

Source: Own illustration.

1.2.2. Building Basis PopGroups (BPGs) and PopGroups (PGs)

Within each MPG, Basis PopGroups (BPGs) were created using empirical analyses in an iterative process based on CDG and MDG assignments and their combinations. In step 1, decision lists with total costs as the target variable were used to identify leading diseases or disease groups within MPGs. In step 2, decision trees were used to identify relevant pairs of leading diseases. In step 3, decision lists as in step 1 were repeated but with the variable list extended by the pairs identified in step 2. The resulting groups were further divided using decision trees based on either the number of distinguishable MDGs in general or the number of severe or very severe CDGs from distinguishable MDGs (as long as the resulting groups met pre-defined criteria, such as a minimum group size). This process resulted in 610 BPGs. Each BPG was subdivided into up to two PopGroups (PGs) using regression trees with healthcare costs as target variable and considering additional individual-level characteristics like age, sex, level of care need, or hours of ventilation (as long as the resulting groups met pre-defined criteria). In total, 776 PopGroups were defined.

1.2.3. Building PopGroup Cost Strata (PGCs)

In a final step, each PopGroup was assigned to one of ten PopGroup Cost Strata (PGCs). These are economically driven segments that group PopGroups by average costs. The PGCs were created so that each stratum accounts for approximately 10% of total healthcare costs.

1.3. PopGroup severity score

The PopGroup severity score represents the relative severity of a PopGroup in comparison to all other PopGroups. Similar to the severity score for CDGs (see section 1.1.2), the relative severity is based on the three dimensions: (1) medical severity, measured by standardized mortality ratios; (2) economic burden, measured by average total costs; and (3) healthcare need, measured by number of average outpatient visits and inpatient days. A standardized composite severity score (z-score) was calculated from these components. Within the healthcare need dimension, inpatient service use was weighted twice in comparison to outpatient service use assuming that hospitalizations reflect more severe conditions. In PopGrouper 1.0, the PopGroup severity score ranges from -0.4 to 13.1. Based on this, PopGroups are ranked by severity and categorized as very severe, severe, moderate, mild, or very mild.

1.4. Examples of PopGroup Assignments

Figure 2 illustrates an example of a PopGroup assignment for a person diagnosed with “Cerebral edema” (ICD-10 G93.6) and “Infection after surgery” (ICD-10 T81.4). This person is assigned to both CDG Z1419 “Cerebral edema” and CDG Z2401 “Complications after surgery”. Since Z1419 is a condition that is both very severe (as determined by the CDG severity score) and among the top 20% costliest conditions the person is grouped into MPG P03 “Severe high-cost cases”. The combination of Z1419 and Z2401 led to the person being assigned to BPG P03016Z “P03: Cerebral edema and complications after surgery”. This BPG is not split at PopGroup level (as none of the additional individual characteristics led to a split that met all pre-defined criteria). That means all persons in BPG P03016Z are also assigned to PopGroup P03016ZZ “P03: Cerebral edema and complications after surgery”. This PopGroup has a relative cost weight of 36.00 (compared to 1.00 for average costs) and is ranked as a very severe PopGroup with a severity score of 6.12.

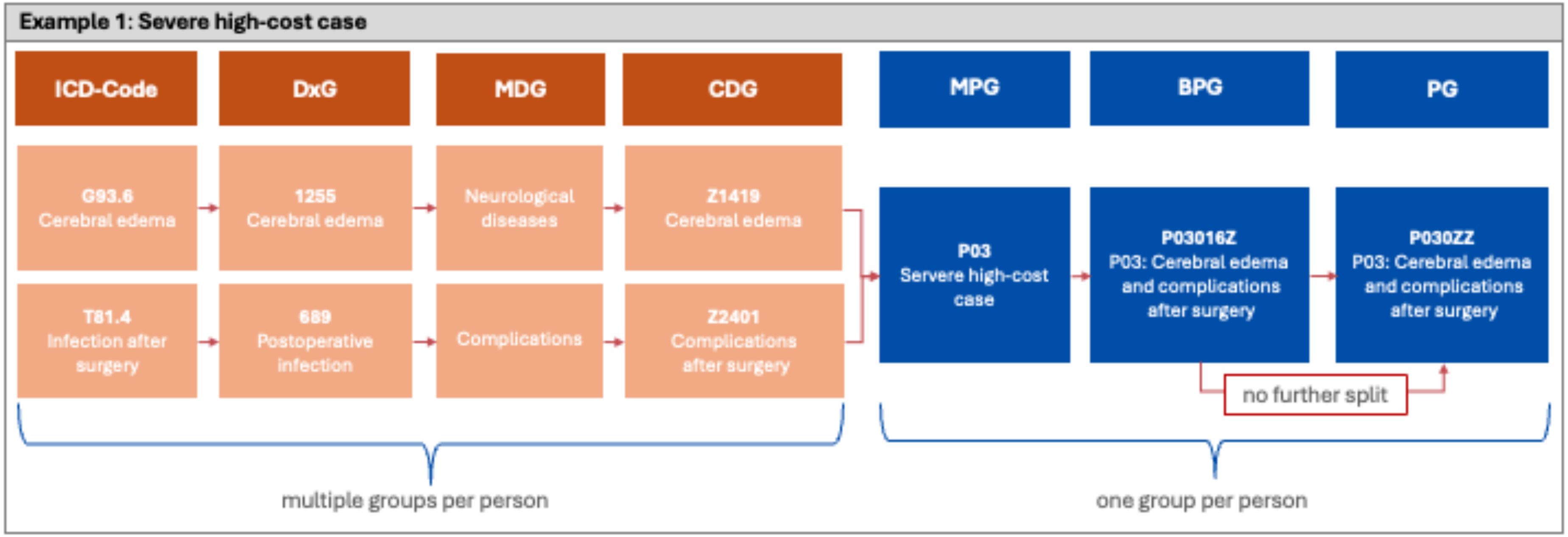

Figure 2: PopGroup assignment example 1 - severe high-cost case

Source: Own illustration.

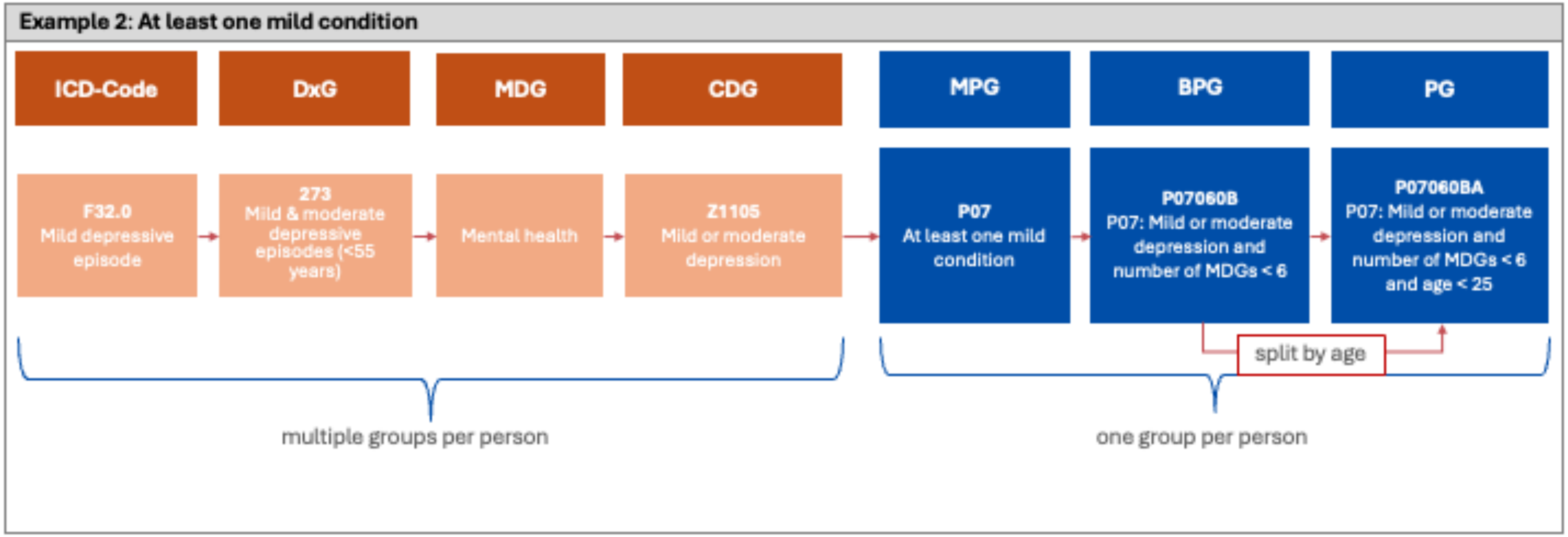

Figure 3: PopGroup assignment example 2 – at least one mild condition

Source: Own illustration.

Figure 3 demonstrates another example of a PopGroup assignment for a person with a mild illness. The person was diagnosed with a mild depressive episode (ICD-10 F32.0) which led to CDG Z1105 “Mild or moderate depression”. Based on the CDG severity score, Z1105 is a mild CDG. Since this individual has no other condition with a higher severity level and does not meet any of the criteria for a higher-ranked MPG (01–06), it is allocated to MPG P07 “At least one mild condition”. Due to the low number of other MDGs, this person is assigned to BPG P07060B “P07: Mild or moderate depression and number of MDGs < 6”. At the PopGroup level, the BPG is split by age with the person being assigned to PopGroup P07060BA “P07: Mild or moderate depression and number of MDGs < 6 and age < 25”. Other diagnoses may be present but are irrelevant to the assignment in this example. This PopGroup has a relative cost weight of 0.69 (compared to 1.00 for average costs) and is ranked as a very mild PopGroup with a severity score of -0.20.

1.5. Validation of the PopGrouper 1.0

The grouping algorithm of PopGrouper 1.0 was developed using 80% of the total BARMER population in the year 2022 as training data. The PopGrouper was then validated using 20% of the total population as test data.

1.6. Background

The PopGrouper was developed as part of a publicly funded research project which ended in September 2024. The grouping algorithm will be made available as open source. The publication of the grouping algorithm is currently in process – updates are announced at www.popgroup.de.

1.7. References

1. Braun A, Grobe TG, Tsatsaronis C et al (2025) [Grouping Algorithm of the PopGrouper Version 1.0]. Working papers in health policy and management. Technische Universität Berlin, Berlin. Available via the institutional repository of Technische Universität Berlin: https:// doi.org/10.14279/depositonce-23953

2. German Federal Office for Social Security (2025) Risikostrukturausgleich: Festlegungen. https://www.bundesamtsozialesicherung.de/de/themen/risikostrukturausgleich/festlegungen/. Accessed 16 Jan 2025

3. Tsatsaronis C, Klemt M, Kinder K, et al (2025) [Definition of consolidated disease groups for a population-based system to classify morbidity-related healthcare needs: PopGroup]. Gesundheitswesen 87:282–290. https://doi.org/10.1055/a-2541-9695
