# Supplementary material for: The PopGrouper as a tool for morbidity adjustment in regional comparisons of health care: an analytical framework
Source: Res Health Serv Reg. 2025 Aug 6;4:10. doi: 10.1007/s43999-025-00068-y (PMC12325817; doi:10.1007/s43999-025-00068-y)
Supplement: Supplementary file 2 — Supplementary Material 2 [file 43999_2025_68_MOESM2_ESM.pdf]

Selection criteria for study populations

Study populations Breast cancer, depression, and type 2 diabetes were selected based on consolidated disease groups (ZKGs) from the PopGrouper version 1.0 [1]. ZKGs build on diagnosis groups (DxGs) used in the German health insurance system to adjust funding received by the health insurers for morbidity [2]. The DxGs are based on ICD-10 codes from the International Statistical Classification of Diseases and Related Health Problems (10th Revision) and additional selection criteria as listed below.

Breast cancer

Persons having at least one of the following ZKGs were included:

ZKG	ZKG title	DxG	DxG title	Selection criteria for DxG classifcation[1]				
				**Age**	**ICD-10 codes**	**Inpatient diagnosis required[2]**	**Medication required[3]**	**Minimum treatment length**
Z0210	Malignant neoplasm of the breast; not actively treated	64	Malignant neoplasm of the breast (Age >44)	>44	C50.0-6, C50.8-9	no	no	no
		78	Malignant neoplasm of the breast (Age <45)	<45	C50.0-6, C50.8-9	no	no	no
Z0238	Malignant neoplasm of the breast; actively treated[4]	64	Malignant neoplasm of the breast (Age >44)	>44	C50.0-6, C50.8-9	no	no	no
		78	Malignant neoplasm of the breast (Age <45)	<45	C50.0-6, C50.8-9	no	no	no

Note: 1 based on German Federal Office for Social Security [2]; 2 if "no", confirmed outpatient diagnosis in at least 2 quarters of the year is sufficient; 3 if "yes", at least 1 simultanuous medication prescription required; 4 selection criteria of Z0210 apply plus additional indicators for actively treated cases which are either an inpatient diagnosis or additional ICD-10 codes Z51.0, Z51.1, Z51.2 or Z51.82 for radiation or chemotherapy.

Depression

Persons having at least one of the following ZKGs were included:

ZKG	ZKG title	DxG	DxG title	Selection criteria for DxG classifcation[1]				
				**Age**	**ICD-10 codes**	**Inpatient diagnosis required[2]**	**Medication required[3]**	**Minimum treatment length**
Z1104	Severe depression	838	Recurrent major depression	-	F33.2-3	no	yes	>=10 days
		839	Severe depression without recurrence	-	F33.2-3	no	yes	>=10 days
Z1105	Mild to moderate depression	271	Recurrent depressive disorder (Age <55)	<55	F33.0; F33.1; F33.4	no	no	no
		272	Recurrent depressive disorder (Age >54)	>54	F33.0; F33.1; F33.4	no	no	no
		273	Mild and moderate depressive episodes (Age <50)	<50	F32.0-1	no	no	no
		274	Mild and moderate depressive episodes (Age >49)	>49	F32.0-1	no	no	no
		476	Other or unspecified affective or neurotic disorders, other neurotic disorders	-	F34.8-9, F38.0-1, F38.8, F39, F48.0, F48.9, F99	no	no	no
		817	Other or unspecified recurrent depressive disorders	-	F33.8-9	no	no	no
		818	Other depressive episodes	-	F32.8-9	no	no	no
		844	Dysthymia	-	F34.1	no	no	no
		1241	Other persistent affective disorders	-	F41.2	no	no	no

Note: 1 based on German Federal Office for Social Security [2]; 2 if "no", confirmed outpatient diagnosis in at least 2 quarters of the year is sufficient; 3 if "yes", at least 1 simultanuous medication prescription required.

Type 2 diabetes

Persons having at least one of the following ZKGs were included:

ZKG	ZKG title	DxG	DxG title	Selection criteria for DxG classifcation[1]				
				Age	ICD-10 codes	Inpatient diagnosis required[2]	Medication required[3]	Minimum treatment length
Z0301	Diabetes mellitus with diabetic ketoacidosis or coma	96	Other diabetes mellitus with diabetic ketoacidosis or coma	-	E11.0; E11.01, E11.1, E11.11, E12.0, E12.01, E12.1, E12.11, E13.0, E13.01, E13.1, E13.11, E14.0, E14.01, E14.1, E14.11	special rule[1]	special rule[1]	special rule[1]
		104	Other diabetes mellitus with diabetic ketoacidosis or coma and long-term insulin medication	-	E11.0, E11.01, E11.1, E11.11, E12.0, E12.01, E12.1, E12.11, E13.0, E13.01, E13.1, E13.11, E14.0, E14.01, E14.1, E14.11, U69.73	yes	no	no
		105	Type 1 diabetes mellitus with diabetic ketoacidosis or coma	-	E10.0, E10.01, E10.1, E10.11	yes	no	no
Z0302	Diabetes mellitus with symptoms in at least one organ(-system)	7	Other diabetes mellitus with kidney involvement and long-term insulin medication	-	E11.2, E11.20, E11.21, E12.2, E12.20, E12.21, E13.2, E13.20, E13.21, E14.2, E14.20, E14.21	special rule[1]	special rule[1]	special rule[1]
		17	Other diabetes mellitus with multiple complications and long-term insulin medication	-	E11.7, E11.72, E11.73, E11.74, E11.75, E12.7, E12.72, E12.73, E12.74, E12.75, E13.7, E13.72, E13.73, E13.74, E13.75, E14.7, E14.72, E14.73, E14.74, E14.75	special rule[1]	special rule[1]	special rule[1]
		54	Other diabetes mellitus with symptoms of the nervous system	-	E11.4, E11.40, E11.41, E12.4, E12.40, E12.41,	special rule[1]	special rule[1]	special rule[1]
		75	Other diabetes mellitus with symptoms of peripheral vessels and long-term insulin medication	-	E11.5, E11.50, E11.51, E12.5, E12.50, E12.51, E13.5, E13.50, E13.51,	special rule[1]	special rule[1]	special rule[1]
		76	Other diabetes mellitus with other specified symptoms and long-term insulin medication	-	E11.6, E11.60, E11.61, E12.6, E12.60, E12.61, E13.6, E13.60, E13.61, E14.6, E14.60, E14.61	special rule[1]	special rule[1]	special rule[1]
		97	Diabetic retinopathy	-	H36.0	no	yes	>=183 days
		98	Other diabetes mellitus with kidney involvement	-	E11.2, E11.20, E11.21, E12.2, E12.20, E12.21, E13.2, E13.20, E13.21, E14.2, E14.20, E14.21	no	yes	>=183 days
		99	Diabetes mellitus type 1 with kidney involvement	-	E10.2, E10.20, E10.21	no	yes	>=183 days
		100	Other diabetes mellitus with symptoms of the nervous system	-	E11.4, E11.40, E11.41, E12.4, E12.40, E12.41,	no	yes	>=183 days
		101	Diabetes mellitus type 1 with symptoms of the nervous system	-	E10.4, E10.40, E10.41	no	yes	>=183 days
		102	Other diabetes mellitus with symptoms of peripheral vessels	-	E11.5, E11.50, E11.51, E12.5, E12.50, E12.51, E13.5, E13.50, E13.51, E14.5, E14.50, E14.51	no	yes	>=183 days
		103	Diabetes mellitus type 1 with symptoms of peripheral blood	-	E10.5, E10.50, E10.51	no	yes	>=183 days
		106	Other diabetes mellitus with other specified symptoms	-	E11.6, E11.60, E11.61, E12.6, E12.60, E12.61, E13.6, E13.60, E13.61,	no	yes	>=183 days
		107	Diabetes mellitus type 1 with other specified symptoms	-	E10.6, E10.60, E10.61	no	yes	>=183 days
		108	Other diabetes mellitus with ocular manifestations	-	E11.3, E11.30, E11.31, E12.3, E12.30, E12.31, E13.3, E13.30, E13.31, E14.3, E14.30, E14.31	no	yes	>=183 days

ZKG	ZKG title	DxG	DxG title	Selection criteria for DxG classifcation[1]				
				Age	ICD-10 codes	Inpatient diagnosis required[2]	Medication required[3]	Minimum treatment length
Z0302	Diabetes mellitus with symptoms in at least one organ(-system)	109	Diabetes mellitus type 1 with ocular manifestations	-	E10.3, E10.30, E10.31	no	yes	>=183 days
		323	Diabetic neuropathy	-	G59.0, G63.2	no	no	no
		430	Diabetic angiopathy	-	I79.2	no	yes	>=183 days
		552	Glomerular Diseases in Diabetes Mellitus	-	N08.3	no	yes	>=183 days
		800	Other diabetes mellitus with multiple complications	-	E11.7, E11.72, E11.73, E11.74, E11.75, E12.7, E12.72, E12.73, E12.74, E12.75, E13.7, E13.72, E13.73, E13.74, E13.75, E14.7, E14.72, E14.73, E14.74, E14.75	no	yes	>=183 days
		801	Diabetes mellitus type 1 with multiple complications	-	E10.7, E10.72, E10.73, E10.74, E10.75	no	yes	>=183 days
		802	Other diabetes mellitus with unspecified complications	-	E11.8, E11.80, E11.81, E12.8, E12.80, E12.81, E13.8, E13.80, E13.81, E14.8, E14.80, E14.81	no	yes	>=183 days
		803	Diabetes mellitus type 1 with unspecified complications	-	E10.8, E10.80, E10.81	no	yes	>=183 days
		1049	Diabetic retinopathy with long-term insulin treatment	-	H36.0	special rule[1]	special rule[1]	special rule[1]
		1050	Diabetic Angiopathy in Long-Term Insulin Medication	-	I79.2	special rule[1]	special rule[1]	special rule[1]
		1051	Diabetic neuropathy in long-term insulin medication	-	G59.0, G63.2	special rule[1]	special rule[1]	special rule[1]
		1052	Glomerular diseases in diabetes mellitus with long-term insulin medication	-	N08.3	special rule[1]	special rule[1]	special rule[1]
		1066	Other diabetes mellitus with ocular manifestations and long-term insulin medication	-	E11.3, E11.30, E11.31, E12.3, E12.30, E12.31, E13.3, E13.30, E13.31, E14.3, E14.30, E14.31	special rule[1]	special rule[1]	special rule[1]
		1067	Other diabetes mellitus with unspecified complications and long-term insulin medication	-	E11.8, E11.80, E11.81, E12.8, E12.80, E12.81, E13.8, E13.80, E13.81, E14.8, E14.80, E14.81	special rule[1]	special rule[1]	special rule[1]
Z0303	Diabetes mellitus without complications	110	Other diabetes mellitus without complications	-	E11.9, E11.90, E11.91, E12.9, E12.90, E12.91, E13.9, E13.90, E13.91, E14.9, E14.90, E14.91, O24.1, O24.2, O24.3	no	no	no
		111	Diabetes mellitus type 1 without complications	-	E10.9, E10.90, E10.91, O24.0	no	yes	>=183 days
		1068	Other diabetes mellitus without complications and long-term insulin medication	-	E11.9, E11.90, E11.91, E12.9, E12.90, E12.91, E13.9, E13.90, E13.91, E14.9, E14.90, E14.91, O24.1, O24.2, O24.3	special rule[1]	special rule[1]	special rule[1]

Note: 1 based on German Federal Office for Social Security [2]; 2 if "no", confirmed outpatient diagnosis in at least 2 quarters of the year is sufficient; 3 if "yes", at least 1 simultanuous medication prescription required.

Congestive heart failure

Since there are some ZKGs from the PopGrouper version 1.0 [1] which combine persons with heart failure and other diseases, we selected this study population based DxGs rather than ZKGs [2]. Persons having at least one of the following DxGs were included:

DxG	DxG title	Selection criteria for DxG classifcation[1]				
		Age	ICD-10 codes	Inpatient diagnosis required[2]	Medication required[3]	Minimum treatment length
354	Hypertensive heart disease, with heart failure	-	I11.0, I11.00, I11.01	no	yes	>=183 days
355	Hypertensive heart / kidney disease, with heart failure	-	I13.0, I13.00, I13.01, I13.00, I13.01	no	yes	>=183 days
367	Severe heart failure (< 75 years)	<75	I50.04, I50.05, I50.13, I50.14	no	yes	>=183 days
368	Severe heart failure (> 74 years)	>74	I50.04, I50.05, I50.13, I50.14	no	yes	>=183 days
369	Secondary right heart failure	-	I50.01	no	yes	>=183 days
376	Mild and unspecified heart failure	-	I50.0, I50.00, I50.02, I50.03, I50.1, I50.11, I50.12, I50.19, I50.9	no	yes	>=183 days
539	Hypertensive kidney / heart disease, with kidney / heart failure	-	I13.2, I13.2, I13.20, I13.20, I13.21, I13.21	no	yes	>=183 days

Note: 1 based on German Federal Office for Social Security [2]; 2 if "no", confirmed outpatient diagnosis in at least 2 quarters of the year is sufficient; 3 if "yes", at least 1 simultanuous medication prescription required.

Acute stroke

For this study population, we aimed to select only persons with an inpatient stay due to an acute stroke event (and no stroke event in the previous year). Hence, we used selection criteria defined in the German Quality Assurance with Routine Data (QSR) procedure which is used to monitor quality outcomes in the German health care system [3]:

Age	ICD-10 codes	Inpatient diagnosis required	Medication required	Minimum treatment length
>30	I61, I63, I64	yes[1]	no	no

Note: 1 main inpatient diagnosis required and no inpatient main diagnosis in the previous 12 months.

References

1. Braun A, Grobe TG, Tsatsaronis C et al (2025) [Grouping Algorithm of the PopGrouper Version 1.0]. Working papers in health policy and management. Technische Universität Berlin, Berlin. Available via the institutional repository of Technische Universität Berlin: https://doi.org/10.14279/depositonce-23953
2. German Federal Office for Social Security (2025) Risikostrukturausgleich: Festlegungen. https://www.bundesamtsozialesicherung.de/de/themen/risikostrukturausgleich/festlegungen/. Accessed 16 Jan 2025
3. Jeschke E, Günster C (2022) Qualitätsindikatoren für stationäre Leistungen: das Verfahren Qualitätssicherung mit Routinedaten (QSR). GG Wiss 25–34
